# The Smoothened agonist SAG Modulates the Male and Female Peripheral Immune Systems Differently in an Immune Model of Central Nervous System Demyelination

**DOI:** 10.3390/cells13080676

**Published:** 2024-04-13

**Authors:** Abdelmoumen Kassoussi, Amina Zahaf, Tom Hutteau-Hamel, Claudia Mattern, Michael Schumacher, Pierre Bobé, Elisabeth Traiffort

**Affiliations:** 1 U1195 Inserm, Paris-Saclay University, 94270 Le Kremlin-Bicêtre, France; 2 UMR996 Inserm, Paris-Saclay University, 91400 Saclay, Francepierre.bobe@universite-paris-saclay.fr (P.B.); 3 MetP Pharma AG, 6376 Emmetten, Switzerland

**Keywords:** oligodendrocyte, microglia, myelination, Hedgehog signaling, androgen, cytokine, NK cell

## Abstract

Both Hedgehog and androgen signaling pathways are known to promote myelin regeneration in the central nervous system. Remarkably, the combined administration of agonists of each pathway revealed their functional cooperation towards higher regeneration in demyelination models in males. Since multiple sclerosis, the most common demyelinating disease, predominates in women, and androgen effects were reported to diverge according to sex, it seemed essential to assess the existence of such cooperation in females. Here, we developed an intranasal formulation containing the Hedgehog signaling agonist SAG, either alone or in combination with testosterone. We show that SAG promotes myelin regeneration and presumably a pro-regenerative phenotype of microglia, thus mimicking the effects previously observed in males. However, unlike in males, the combined molecules failed to cooperate in the demyelinated females, as shown by the level of functional improvement observed. Consistent with this observation, SAG administered in the absence of testosterone amplified peripheral inflammation by presumably activating NK cells and thus counteracting a testosterone-induced reduction in Th17 cells when the molecules were combined. Altogether, the data uncover a sex-dependent effect of the Hedgehog signaling agonist SAG on the peripheral innate immune system that conditions its ability to cooperate or not with androgens in the context of demyelination.

## 1. Introduction

Multiple sclerosis (MS) is a chronic, inflammatory disease resulting in demyelination and neuronal degeneration in the central nervous system (CNS) [1]. Genetic predisposition and environmental factors likely contribute to an improper immune response initiated in the periphery, leading to the activation of autoreactive B and T cells, which infiltrate the CNS [2]. In most cases, the disease begins with a succession of demyelination episodes, each followed by the spontaneous regeneration of myelin and reflected by the onset of transient neurological disabilities. This step of the disease is called relapsing-remitting MS. However, over time, inescapable progression leads to irreversible disabilities related to the failure of myelin regeneration, which contributes to axonal damage and neuronal death, characterizing the secondary progressive MS [3]. Drugs acting on the inflammatory component of the disease have been used over the last few decades and have led to an efficient reduction in the frequency and severity of the relapses [4,5,6,7]. Even though they slow down the appearance of new demyelinating lesions, the impact of these drugs on disease progression remains nevertheless unsatisfactory. Therefore, boosting myelin regeneration to confer neuroprotection is the current challenge in the field, especially if we consider the strong inverse correlation evidenced between myelin regeneration and disability levels [8].

We previously identified a drug combination endowed with the capacity to break the vicious circle of ‘inflammation-demyelination-neurodegeneration’ that, up to now, could not be halted by currently available treatments [9]. This combination relies on the simultaneous activation of two positive regulators of spontaneous myelin regeneration, the Hedgehog and androgen signaling pathways. On the one hand, in the model of focal demyelination of the corpus callosum, the blocking of the secreted protein Sonic Hedgehog by its physiological antagonist, Hedgehog interacting protein, was found to prevent myelin regeneration by decreasing the proliferation and differentiation of oligodendrocyte progenitor cells (OPCs) and by impeding in microglial cells the up-regulation of the Smoothened (Smo) receptor, one of the key mediators of Hedgehog signaling [10]. On the other hand, the male sexual hormone, testosterone, and the androgen receptor AR appeared to be critical in astrocyte recruitment and spontaneous oligodendrocyte-mediated myelin regeneration upon focal demyelination of the ventral funiculus of the spinal cord [11]. When the androgen and Hedgehog pathways are simultaneously activated after focal demyelination using testosterone and the well-characterized Smo agonist, SAG, higher myelin regeneration was observed compared to the administration of each drug used alone, suggesting their functional cooperation. Furthermore, the combined drugs additionally led to the higher preservation of axon integrity, lower neuroinflammation, and almost complete regression of neurological disabilities in animals demyelinated using the experimental autoimmune encephalomyelitis (EAE) model. In the latter, the molecular mechanism supporting this functional cooperation appeared to uniquely involve the collapse of deleterious cytokine levels, including GM-CSF, TNF-α, and IL-17A, in the demyelinated CNS [9]. If we consider the current idea that the compartmentalized CNS immune reactions are proposed to be involved in MS progression instead of peripheral immune dysregulation, which is known to guide the relapsing-remitting form of the disease [8,12,13], the improvement of myelin regeneration and lowering of neuroinflammation induced by this drug combination are likely important to take into account in the context of secondary progressive MS.

However, these data regarded only male animals. Since we recently reported that androgens show sex-dependent differences in myelination in immune and non-immune murine models of CNS demyelination [14], it is questionable whether this drug combination has comparable effects in female animals. To this purpose, we assessed the molecules alone or in combination in female mice demyelinated using immune and non-immune demyelination models. We show that, in females, the Smo agonist SAG has a pro-myelinating activity comparable to the one previously observed in males in a demyelination context devoid of major peripheral immune system deregulation. Conversely, SAG does not cooperate with testosterone in the context of immune-mediated demyelination in females, unlike in males. SAG is indeed endowed with a peripheral pro-inflammatory activity in females devoid of androgens, an effect likely related to its ability to increase the proportion of natural killer (NK) cells abrogated by exogenous testosterone administration and nevertheless preventing any functional cooperation. This finding uncovers that SAG activity is conditioned by testosterone levels in females. Furthermore, it highlights the largely underestimated concept of the need to take into account the sex of the animal in determining the effect of individual or combined drugs and a fortiori the sex of the patient to be treated. 

## 2. Materials and Methods

**Animals.** Wild-type gonadectomized C57BL/6 female mice were purchased at the age of 8 to 12 weeks from Janvier Labs Breeding Center (France). All animals were housed in standard conditions: a 12 h light–dark cycle with food and water ad libitum. All procedures were performed according to the European Communities Council Directive (86/806/EEC) for the care and use of laboratory animals and were approved by the Regional Ethics Committee CEEA26, Ministère de l’Education Nationale, de l’Enseignement et de la Recherche. 

**Drugs.** The Smo agonist SAG [15] was purchased from D&C Chemicals and Shanghai Haoyuan Chemexpress (Shanghai, China). Testosterone was provided by Pharmacia & Upjohn (Kalamazoo, MI, USA). The oral administration of SAG (15 mg/kg) was performed after the dissolution of the molecule in methylcellulose (0.5%)/Tween80 (0.2%) all provided by Sigma-Aldrich, Saint Quentin Fallavier, France. The intranasal administration used testosterone (0.2 mg/day) as previously described [16], and SAG was dissolved in aqueous solution and adsorbed on silica. The intranasal gels were then prepared by mixing Labrafil M 1944 CS (Gattefossé, Neuilly-sur-Seine, France), castor oil (Gustav Heess, Leonberg, Germany), Aerosil 200 (Evonik, Berlin, Germany) and the silica-SAG particles to obtain gels containing 2%, 4% and 6% of SAG, respectively, i.e., 0.1, 0.2 and 0.3 mg/day under a volume of 2.5 µL in each nostril. The SAG/testosterone combination gel containing 6% SAG and 4% testosterone were prepared in the same way, except that testosterone was first dissolved in the castor oil (all gels were patented proprietary developments of MetP Pharma AG, Emmetten, Switzerland).

**LPC-induced focal demyelination.** The demyelination of the right corpus callosum was performed by stereotaxic injections of LPC at 1%, as previously described [16]. The demyelinated tissues were analyzed at 7 (*n* = 3–5 mice/condition) or 10 (*n* = 4 mice/condition) days postlesion (dpl) after animal perfusion, tissue post-fixation (PFA 4%) and cryopreservation (30% sucrose) before freezing and cryostat sectioning (14 µm).

**Autoimmune Experimental Encephalomyelitis.** Ovariectomized female mice at the age of 9–10 weeks were used after one week of acclimatization. The pathology was induced by the subcutaneous injection of an emulsion of the MOG_35–55_ peptide in complete Freund’s adjuvant, as previously described [17]. Vehicle, SAG, testosterone, or SAG-testosterone treatments were administered at the onset of neurological disabilities (*n* = 10 animals per group) and evaluated blindly until Day 30 post-immunization according to the classical scale, as described [14]. Drugs or the vehicles were administered daily via the intranasal route at the onset of clinical symptoms until Day 30 or Day 14 after immunization. The spinal cord was harvested, post-fixed (PFA 4%) for 24 h, and sectioned (7 µm) using a microtome before immunostaining (*n* = 4 mice/condition).

**Immunostaining experiments.** The primary antibodies used for the immunostaining experiments were those previously described [9] including Olig2 (rabbit, mouse, 1:500, AB9610, MABN50, Millipore, Burlington, MA, USA/Sigma Aldrich, St. Louis, MO, USA), PDGFRα (rat, 558774, 1:500, BD Pharmingen, Le Pont de Claix, France), Ki67 (mouse, 550609, 1:100, BD Pharmingen, Adenomatus Polyposis Coli (APC/CC1) (mouse, OP80, 1:500, Calbiochem, Sigma Aldrich), PLP (mouse, Mab388, 1:250, Millipore), MBP (rabbit, AB980, 1:1000, Millipore), Iba1 (rabbit, W1 W019-19741, 1:500, Wako/Sobioda, Montbonnot Saint Martin, France), GFAP (Rabbit, ZO334, 1:1000, Dako/Agilent technologies, Les Ulis, France; mouse, G3893, 1:1000, Sigma), Arg-1 (goat, sc-18355, 1:100, Santa-Cruz/Bio-Techne, Noyal Chatillon sur Seiche, France), and non-phosphorylated Smi-32 (mouse, 801701, 1:500, Biolegend, London, UK). Secondary antibodies included goat anti-rabbit cyanine 3 conjugated (111 165 003, 1/250, Jackson Immunoresearch/Interchim, Montluçon, France); goat anti-mouse Alexa 488 (A11029, 1:250), anti-rabbit Alexa 633 (A21070, 1:750), anti-rat Alexa 633 (A21094, 1:750), anti-chicken Alexa 546 (A11040, 1:250), donkey anti-goat Alexa 546 (A11056, 1:250) provided by Thermo Fisher Scientific (Les Ulis, France) and goat anti-chicken Alexa 488 (Ab150169, 1:750) provided by Abcam (Paris, France).

**Image Acquisition and Analysis.** Axiovision 4.2 (Carl Zeiss, Inc., Oberkochen, Germany), as well as the confocal Zeiss LSM 510-Meta Confocor 2 and the scanner imager (Model Pannoramic 250 Flash II Marque 3DHISTECH), were used together with the ImageJ win64 v1.53 software. A minimum of 10 sections per animal were analyzed. One out of every five sections was immunostained throughout the LPC demyelinated lesion for quantifying the immunofluorescent cells or measuring the immunofluorescent areas. The lesion surface was evaluated by measuring the area in which nuclear densification was observed and well correlated with myelin loss visualized by MBP or PLP staining.

**Flow cytometry.** Immune cells from the lymph nodes and spleen (*n* = 6 mice/condition) were phenotyped by flow cytometry using the fluorescent-conjugated antibodies previously described [9], including CD90.2/Thy1.2 (clone 30-H12), B220 (clone RA3-6B2), CD45RB (clone C363.16A), CD4 (clone GK1.5), CD8α (clone 53-6.7), CD44 (clone IM7), Ly6G (clone RB6-8C5), F4/80 (clone BM8), NK1.1 (clone PK 136), and CD11c (clone N418). The transcription factors Foxp3, T-bet, and RORγt were recognized by clones FJK-16s, eBio4B10, and B2D, respectively, provided by BD Biosciences or eBioscience Thermo Fisher Scientific. Non-specific antibody binding was prevented using the mouse Fcγ receptor (clone 93, eBioscience Thermo Fisher Scientific). Data were acquired at the flow cytometry core facility at IPSIT, Université Paris-Saclay, INSERM, CNRS (Clamart, France) and analyzed using the FlowJo v10.8.1 software (Treestar, Ashland, OR, USA).

**Quantification of cytokines.** Cytokine levels were determined using the Bio-Plex Pro Mouse cytokine 8-plex Assay (Bio Rad M60000007A) and cytokine IL-17A Set (Bio Rad, Hercules, CA, USA, 171G5013M), as previously described [9], on cell lysates (*n* = 6 mice/condition) containing similar amounts of proteins as determined using the Pierce BCA Protein Assay Kit (Thermo Fisher Scientific). Data acquisition was performed on a Luminex MAGPIX System.

**Statistical analysis.** Statistical analyses were performed using GraphPad Prism 7.0 software (La Jolla, CA, USA). Unpaired and two-tailed Student’s *t*-tests were used for two independent group comparisons. Comparisons of more than two groups or several variables were performed using ANOVA followed by Tukey’s post-tests. Any absence of normal distribution led to the use of non-parametric tests (Mann–Whitney two-tailed, Kruskal–Wallis with Dunn’s post-tests for comparison). Adequate corrections were performed in the case of unequal variances. The values are the means ± SEM from the number of animals indicated in each plotted graph or as indicated in the corresponding legends. A significance of *p* < 0.05 was used for all analyses. * *p* ≤ 0.05; ** *p* ≤ 0.01; *** *p* ≤ 0.001; **** *p* < 0.0001.

## 3. Results

### 3.1. An Innovative Intranasal Formulation of the Smo agonist SAG Is Endowed with an Efficiency Similar to Its Oral Delivery

The intranasal route is a non-invasive method for drug administration. Previously, we validated the intranasal administration of testosterone as a privileged delivery of the hormone to the CNS in both males and females [9,14]. Therefore, it appeared interesting to also assess this administration route instead of the oral route previously used for the Smo agonist SAG, hence the need to determine the dose of intranasal SAG leading to an effect comparable to the oral administration. We induced CNS demyelination in ovariectomized female animals via the stereotaxic injection of LPC into the corpus callosum and analyzed the demyelinated area at 7 days post-lesion (dpl). Six groups of animals received either a daily intranasal administration of SAG at 2%, SAG at 4%, SAG at 6% or the corresponding drug vehicle starting 15 h after LPC injection or a daily oral administration of SAG (15 mg/kg) compared to the corresponding drug vehicle. Then, we evaluated SAG effects in brain slices using immunostaining experiments. The objective was to determine if intranasal SAG could impact the response of microglia and oligodendrocyte progenitor cells (OPCs) to demyelination in the same way as SAG administered via the oral route. 

**Figure 1:**
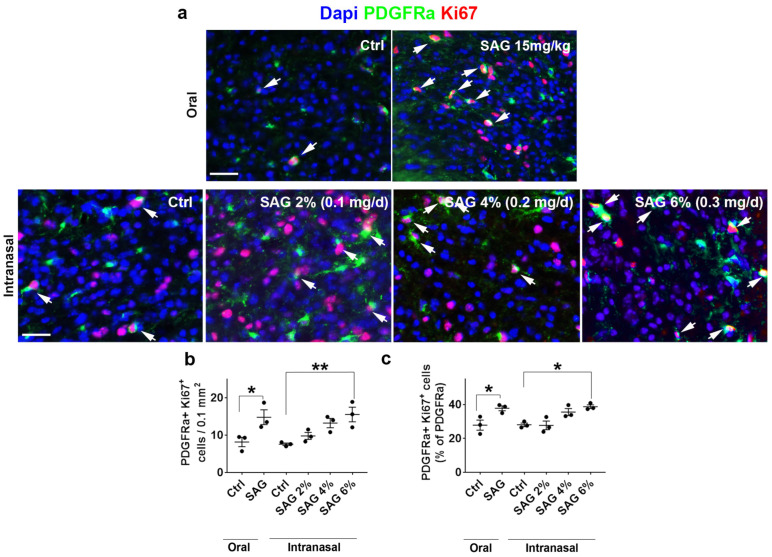
Intranasal SAG controls the response of OPCs to demyelination at 7 days post-lesion in the LPC model. (**a**) PDGFRα and Ki67 immunostaining at the level of the demyelinated corpus callosum from ovariectomized female mice treated with oral (**top panel**) or nasal (**bottom panel**) SAG or athe vehicle. The white arrows indicate cells co-expressing PDGFRa and Ki67 markers. (**b**,**c**) Histograms showing the quantifications of PDGFRα and Ki67-expressing cells in the demyelinated area. Student’s and ANOVA tests are used for the statistical analysis of SAG preparations compared to their respective controls. * *p* ≤ 0.05; ** *p* ≤ 0.01. Scale bars: 50 µm.

At 7 dpl, in females, oral SAG increases the number of OPCs that proliferate in the demyelinated area, as indicated by immunostaining using PDGFRα as a marker of OPCs and Ki67 as a marker of proliferation. Oral SAG increased the number (Figure 1a(top panel),b) and the percentage (Figure 1a(top panel),c) of proliferating OPCs as previously observed in castrated males. The intranasal gels containing SAG at a concentration of 2, 4 or 6% led to an effect depending on the dose. Only SAG at 6% was able to significantly increase both the total density (Figure 1a(bottom panel),b) and the proportion (Figure 1a(bottom panel),c) of proliferating OPCs. 

**Figure 2:**
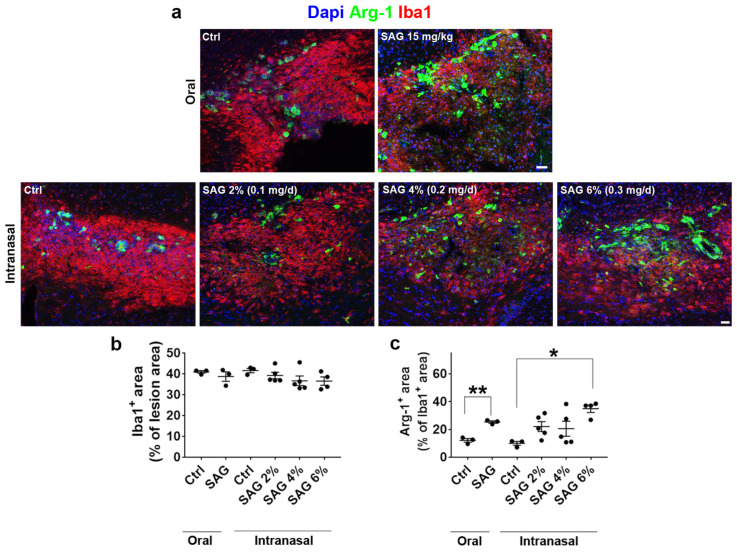
Intranasal SAG controls the response of microglia to demyelination at 7 days post-lesion in the LPC model. (**a**) Iba1 and Arg-1 immunostaining at the level of the demyelinated corpus callosum from ovariectomized female mice treated with oral (**top panel**) or nasal (**bottom panel**) SAG or athe vehicle. (**b**,**c**) Histograms reflecting the quantifications of Iba1 and Arg-1-expressing areas in the demyelinated area. Student’s and ANOVA tests are used for the statistical analysis of SAG preparations compared to their respective controls. * *p* ≤ 0.05; ** *p* ≤ 0.01. Scale bars: 50 µm.

Then, we analyzed oral SAG efficiency to control the lesion area occupied by the whole Iba1+ microglial population or by the microglia subset co-expressing the anti-inflammatory marker Arg-1. As previously shown in males [9], oral SAG did not modify the Iba1^+^ area, whereas it increased Arg-1 expression in the lesion in females (Figure 2a(top panel),b). Intranasal SAG displayed a similar activity profile. However, again, only the highest dose, SAG at 6%, induced a significant increase in the subset of microglia expressing the anti-inflammatory marker (Figure 2a(bottom panel),c). Thus, regardless of its route of administration, SAG did not control the level of microgliosis in females, but only its capacity to express or not the anti-inflammatory marker Arg-1, as previously demonstrated in castrated males using oral SAG [9].

Altogether, these data show that intranasal SAG 6% is as efficient as oral SAG in controlling the proliferation of OPCs and the response of microglia to CNS demyelination. Therefore, SAG 6% appeared to be the dose to be used in future experiments.

### 3.2. Intranasal SAG Promotes OPC Differentiation and Increases PLP Expression in LPC Demyelinated Lesions from Female Mice

**Figure 3:**
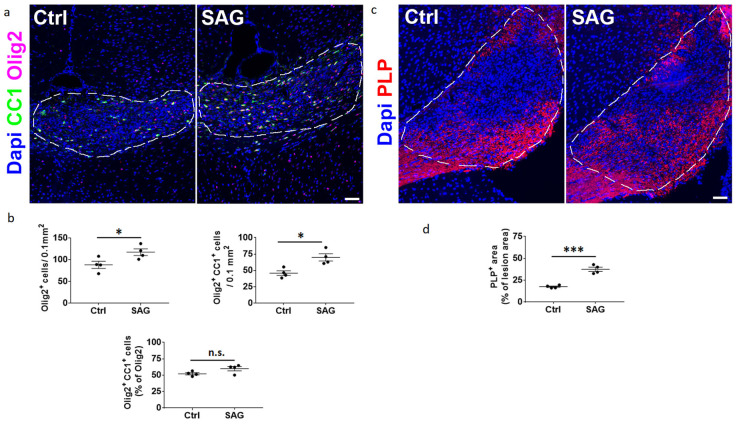
Intranasal SAG increases the differentiation of OPCs into CC1+ oligodendrocytes and promotes PLP expression at 10 days post-lesion in the LPC model. Olig2/CC1 (**a**,**b**) and PLP (**c**,**d**) immunostaining and quantification in the demyelinated corpus callosum from ovariectomized female mice treated with intranasal SAG at 6% or a vehicle (Ctrl). The dashed lines in a and c delineate the demyelinated area. Student’s *t*-test was used for statistical analysis. * *p* ≤ 0.05; *** *p* ≤ 0.001; n.s., not significant. Scale bars: 100 µm.

In order to further characterize intranasally delivered SAG activity, we induced CNS demyelination in ovariectomized female animals via the stereotaxic injection of LPC into the corpus callosum and analyzed the demyelinated area at 10 dpl. Two groups of animals received either a daily intranasal administration of SAG 6% or the corresponding drug vehicle starting 15 h after LPC injection. SAG at 6% significantly increased the total number of CC1+ differentiated oligodendrocytes (69.9 ± 5.5 vs. 45.8 ± 3.6; *p* = 0.01), while the percentage of differentiated oligodendrocytes among the whole population of Olig2+ oligodendroglial cells was not significantly modified (59.8 ± 1.0 vs. 52.2 ± 2.5; Figure 3a,b). We also evaluated the ability of SAG to promote the maturation of CC1+ cells into myelinating cells expressing the PLP protein. The quantification of the PLP+ area in the percentage of the lesion area was significantly higher in the SAG condition compared to the vehicle condition (37.4 ± 3.6 vs. 17.5 ± 0.3; *p* = 0.0002; Figure 3c,d). Thus, intranasal SAG at 6% increases the density of differentiated oligodendrocytes, which subsequently increases remyelination in the demyelinated area.

### 3.3. SAG and Testosterone Both Mitigate the Neurological Disabilities Occurring in EAE Female Mice without Any Additive or Synergistic Effects

Based on the cooperative effects previously reported between SAG and testosterone in male mice demyelinated via immunization against the MOG_35–55_ peptides, we addressed the question of the existence of similar cooperation between intranasal SAG and intranasal testosterone in females using the EAE model considered as a translational model of multiple sclerosis. EAE was induced in adult ovariectomized female mice by the subcutaneous injection of an emulsion of MOG_35–55_ peptide in complete Freund’s adjuvant, as previously described [9,14]. Mice developing EAE were assigned daily to one of the four treatments: vehicle, intranasal testosterone (0.2 mg), intranasal SAG 6% (0.3 mg), and the combination of testosterone and SAG (at the same doses, respectively). Drug treatments started at the onset of neurological signs (day 7 post-immunization = treatment day 1), corresponding to a therapeutic rather than a preventive approach, and were administered for 24 days, when spinal cords were sampled for histological analysis. During the experiment, mice were scored blindly according to the commonly used EAE disease scale every day, starting at treatment day 1 until treatment on day 24.

**Figure 4:**
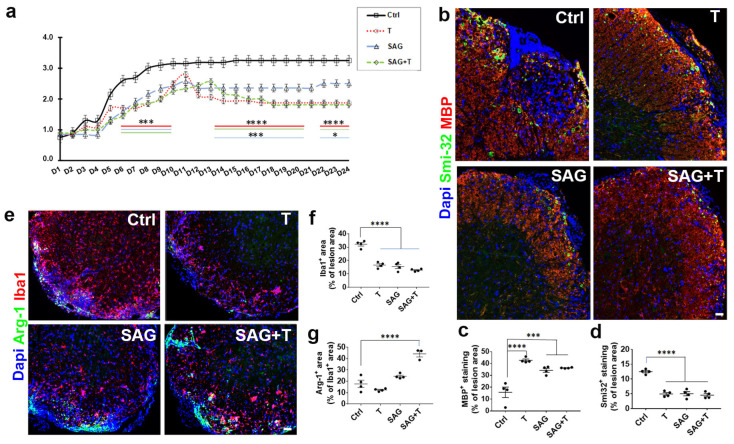
Limited cooperation between SAG and testosterone in the female EAE model. (**a**) The therapeutic administration of testosterone or SAG separately or in combination mitigates the course of EAE in female mice without showing any cooperation. The functional scores are derived from EAE ovariectomized female mice treated with the drug vehicle (Ctrl, black), testosterone (T, red), the Smo agonist (SAG, blue) or the combined drugs (SAG + T, green)) from the onset of the first neurological symptoms (day 1) until day 24 (two-way ANOVA: treatment: F(3, 669) = 281, *p* < 0.0001; time: F(23, 669) = 76,12, *p* < 0.0001). At treatment day 24, visualization of MBP/Smi-32 (**b**–**d**) and Iba1/Arg-1 (**e**–**g**) immunostaining and quantifications occurred in the spinal cord from the ovariectomized EAE female mice. Each treatment condition increases and decreases MBP- and Smi-32 expression, respectively. Testosterone amplifies the positive effect induced by SAG on the anti-inflammatory marker Arg-1. ANOVA tests are used for statistical analysis. * *p* ≤ 0.05; *** *p* ≤ 0.001; **** *p* ≤ 0.0001. Scale bars: 50 µm.

Vehicle-treated animals displayed the typical profile of disease progression with hindlimb paralysis reflected by a 3.2 clinical score reached by treatment day 8 and persisting until the end of the experiment. Animals receiving testosterone, SAG, or their combination progressively increased until reaching the 2.5 scores between treatment days 1 and 10. While SAG maintained this score until the end of the experiment, testosterone (T), either alone or in combination with SAG, reached a plateau at a lower level of around 1.8 (Figure 4a), reflecting a result differing from the one previously evaluated in males [9].

### 3.4. The Combination Therapy Does Not Improve Myelin Status and Axonal Integrity More Effectively Than Administering Each Molecule Separately

We determined MBP and Smi-32 immunostaining to evaluate myelin status and axonal damage, respectively, in the treated females. MBP expression was significantly increased in the presence of each drug condition (T: 42.7 ± 1.1, *p* < 0.0001; SAG: 32.1 ± 1.1, *p* = 0.0017; SAG + T: 39.3 ± 0.3, *p* < 0.0001) compared to the vehicle condition (15.7 ± 4.4). No significant difference was observed between the various drug conditions (Figure 4b,c). All drug conditions also significantly and similarly decreased the expression of Smi-32 (T: 4.9 ± 0.5, *p* < 0.0001; SAG: 5.0 ± 0.7, *p* < 0.0001; SAG + T: 4.5 ± 0.5, *p* < 0.0001) compared to the vehicle condition (12.5 ± 0.4; Figure 4b,d).

### 3.5. SAG Administered Alone or in Combination with Testosterone Maintains Microglia in a Pro-Regenerative Phenotype 

The drugs decreased the expression of the Iba1 microglial marker in a comparable manner (Figure 4e,f; T: 16.6 ± 1.1, *p* < 0.0001; SAG: 15.5 ± 1.5, *p* < 0.0001; SAG + T: 12.8 ± 0.5, *p* < 0.0001) compared to the vehicle condition (32.1 ± 1.3). On the contrary, the effect of the drugs on the expression of the anti-inflammatory marker Arg-1 varied according to the treatment (Figure 4e,g). Indeed, as expected from the lower level of disabilities and a higher level of MBP expression, T decreased Arg-1 (10.3 ± 0.2; *p* = 0.05) compared to the vehicle condition (17.7 ± 3.2). However, despite the lower levels of disabilities and higher expression of MBP observed in the two other groups than in the controls, SAG increased Arg-1 (24.4 ± 1.0; *p* = 0.05) while SAG + T highly increased (44.2 ± 2.7, *p* < 0.0001) this anti-inflammatory marker, fully contrasting the latter condition with data previously reported in males [9].

### 3.6. SAG-Induced Increase in Cytokine Levels Is Reversed by Testosterone

**Figure 5:**
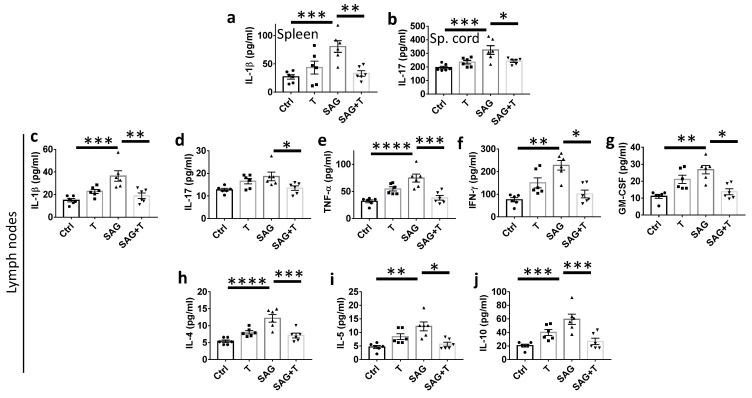
Cytokine secretion in the EAE animals. Cytokine levels were determined in EAE females after the therapeutic administration of SAG and testosterone (T) used separately or simultaneously compared with vehicle (Ctrl) administration for 9 days from the onset of neurological disabilities in the spleen (**a**), spinal cord (**b**) and lymph nodes (**c**–**j**). Data are the mean ± SEM from the number of animals indicated in the plotted graphs. * *p* ≤ 0.05; ** *p* ≤ 0.01; *** *p* ≤ 0.001; **** *p* ≤ 0.0001.

We then investigated whether the cytokine profiles in the spleen, lymph nodes, and spinal cord may be correlated with the respective functional effects of T, SAG, and SAG + T. To this purpose, we harvested tissues from animals treated with the drugs or vehicle for 9 days after the onset of the neurological disabilities at the peak of the functional scores (Figure S1a). We evaluated the expression of Claudin-5, a tight junction protein of the blood–brain barrier, which is vital for maintaining the integrity of endothelial cells lining the brain blood vessels. Claudin-5 expression was significantly increased in all treatment conditions compared to the vehicle conditions, indicating the capacity of the drugs alone or in association to preserve the blood–brain barrier integrity (Figure S1b,c). We determined the levels of the pro-inflammatory (IL-1β, IL-17A, GM-CSF, IFN-γ, TNF-α) and anti-inflammatory (IL-4, IL-5, IL-10) cytokines. Compared to the vehicle, SAG induces a significant increase in the pro-inflammatory molecules IL-1β (80.6 ± 10.3 vs. 26.0 ± 4.0; *p* = 0.0010) and IL-17A (324.4 ± 31.8 vs. 194.8 ± 10.2; *p* = 0.0004) in the spleen and spinal cord, respectively (Figure 5a,b). In the lymph nodes, all the pro-inflammatory cytokines were increased by the SAG treatment (Figure 5c–g), as shown for IL-1β (36.4 ± 4.6 vs. 15.0 ± 1.5; *p* = 0.0002), IL-17A (18.7 ± 1.9 vs. 12.7 ± 0.6; *p* = 0.025), TNF-α (74.5 ± 7.3 vs. 31.9 ± 2.7; *p* < 0.0001), IFN-γ (228.9 ± 21.4 vs. 75.7 ± 9.4; *p* = 0.0021), GM-CSF (26.8 ± 2.5 vs. 11.1 ± 1.2; *p* = 0.0038). Along the same lines, all anti-inflammatory cytokines were also increased by SAG (Figure 5h–j), as determined for IL-4 (12.2 ± 1.1 vs. 5.4 ± 0.4; *p* < 0.0001), IL-5 (12.2 ± 1.6 vs. 4.6 ± 0.6; *p* = 0.0025), IL-10 (59.4 ± 7.5 vs. 20.5 ± 2.2; *p* = 0.0001). Importantly, testosterone was able to reverse SAG effects whether the cytokine was pro- or anti-inflammatory. Indeed, in SAG + T animals, the levels of IL-1β (*p* = 0.0018), GM-CSF (*p* = 0.03), IFN-γ (*p* = 0.03), TNF-α (*p* = 0.0002), but also IL-4 (*p* = 0.0005), IL-5 (*p* = 0.0187), IL-10 (*p* = 0.0010) were significantly decreased compared to the SAG condition, appearing not significantly different from the control condition; this indicates that SAG is endowed with an unexpected pro-inflammatory effect in females devoid of androgens, with nevertheless a restricted impact on the inflammatory status of the spinal cord.

### 3.7. SAG Regulates the Peripheral Innate Immune Cells towards a Pro-Inflammatory Phenotype

**Figure 6:**
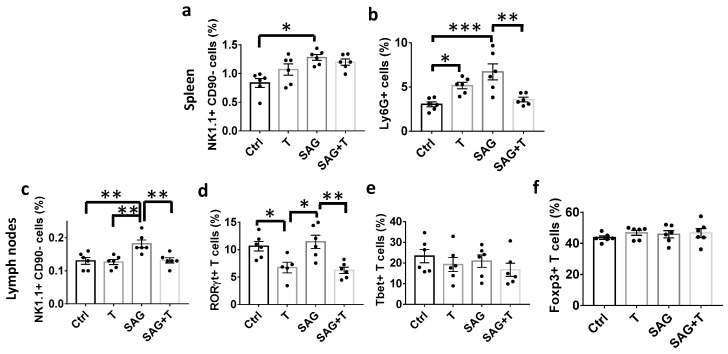
Immunophenotyping of myeloid and lymphoid cells from the lymphoid organs. Flow cytometry analyses were performed on cells isolated from the spleen and inguinal lymph nodes derived from EAE animals treated for 9 days with the indicated drugs from the onset of neurological disabilities onward. The indicated myeloid (**a**–**c**) and lymphoid (**d**–**f**) cells were analyzed according to the gating strategies described in Figures S2–S5. * *p* ≤ 0.05; ** *p* ≤ 0.01; *** *p* ≤ 0.001.

Finally, we explored immune cell subsets present in the spleen and in the CNS-draining lymph nodes, which are known to be essential for the balancing of protective or tolerogenic versus detrimental responses in the CNS [18]. The phenotype and activation status of the main immune cells were assessed by flow cytometry, and cells were gated, as shown (Figures S2–S5). Regarding the myeloid lineage, the NK1.1^+^ CD90^−^ natural killer (NK) cells were significantly increased by SAG in both the spleen (1.28 ± 0.05 vs. 0.83 ± 0.08; *p* = 0.0086; Figure 6a) and the lymph nodes (0.18 ± 0.01 vs. 0.13 ± 0.01; *p* = 0.0053; Figure 6c) whereas the SAG effect was reversed in the presence of testosterone only in the lymph nodes (0.13± 0.01; *p* = 0.007; Figure 6c). In the spleen, the SAG-mediated increase in Ly6G+ granulocytes was also observed (6.7 ± 0.9 vs. 3.0 ± 0.3; *p* = 0.0004) and reversed in the presence of testosterone (3.6 ± 0.3; *p* = 0.0023; Figure 6a). Regarding the lymphoid lineage, testosterone (6.7 ± 1.0, *p* = 0.04) and the combined drugs (6.2 ± 0.6, *p* = 0.01) significantly decreased the percentage of the effector Th cell subsets expressing the ROR-γt (Th17) transcription factor compared to the controls (10.6 ± 0.9; Figure 6d) in the lymph nodes. In contrast, among the CD44^hi^CD45RB^lo^ effector CD4^+^ T cells, neither the Th cell subset expressing the T-bet transcription factor (Th1; Figure 6e) nor the Th subset expressing the Foxp3 transcription factor (regulatory T cells; Figure 6f) were significantly regulated by any of the tested conditions. Altogether, these data indicate that testosterone and SAG control the peripheral immune cells in opposite ways since the former specifically decreases the effector Th cell subset Th17, whereas SAG increases both NK cells and granulocytes.

## 4. Discussion

The questions addressed in the present work were whether the Hedgehog and androgen signaling pathways, previously shown to cooperate in CNS demyelination models in male animals, may similarly act in females. To this end, we formulated SAG in order to be administered via the intranasal route and used two models of demyelination. Upon focal demyelination of the corpus callosum, SAG appeared to be as effective in females as previously shown in males [9]. Indeed, intranasal SAG at 6%, like oral SAG at 15 mg/kg, was able to increase OPC proliferation and promote the expression of the anti-inflammatory marker Arg-1 in microglia. In addition, intranasal SAG increases the density of differentiated oligodendrocytes as well as PLP expression in the demyelinated area, indicating that in females as in males, the activation of the G protein-coupled receptor Smo, the key mediator of Hedgehog signaling, increases the expression of myelin proteins following an increase in the number of proliferating OPCs. However, while the SAG-induced regulation of Arg-1 expression was found to result in a higher percentage of OPCs able to differentiate into CC1+ oligodendrocytes in males [9], SAG fails to increase the proportion of differentiating OPC in females. This observation may suggest that in females, the control of the Arg-1 anti-inflammatory marker is likely, not sufficient by itself to induce a pro-regenerative phenotype of microglia or, alternatively, that SAG is endowed with additional activities possibly counteracting these pro-differentiating effects. Moreover, our data also indicate that the intranasal route is an alternative route that should be considered for the administration of the Smo agonist SAG, which has otherwise been reported to have potential as a neuroprotective agent, such as in neonates at risk for glucocorticoid-induced neonatal cerebellar injury [19].

Although SAG appears to be pro-myelinating regardless of the animal sex in the non-immune LPC-induced model of demyelination, the situation is only partially comparable in males and females in the context of demyelination triggered by a deregulated immune response. In the female EAE model, SAG administration is indeed associated with an increase in MBP expression compared to the vehicle condition in the demyelinated spinal cord, as previously shown in males [9]. This higher myelin status may be the consequence of SAG’s effect on OPC proliferation, as shown above in the LPC model, in agreement with data obtained in vitro in culture experiments [9] and/or on the integrity of the blood–brain barrier suggested by the higher level of Claudin-5 known to be critical for maintaining the integrity of the endothelial cells lining the brain blood vessels. The latter hypothesis is consistent with data also reported in female mice indicating that Smo-mediated Hh pathway activation is used by perivascular astrocytes to communicate with the endothelial cells comprising the blood–brain barrier to promote its repair and also to counter-balance inflammatory events induced during lesion formation [20]. Thus, we can expect SAG to restore physiological and immunological blood–brain barrier competence in EAE animals since SAG similarly regulates Claudin-5 in EAE male [9] and female (present data) mice. Myelin status improvement, together with blood–brain barrier preservation, is consistent with both the lower level of axonal damage and the lower disability scores detected in the SAG-treated EAE males and females. 

Remarkably, the beneficial activity of SAG at the blood–brain barrier and in the nervous parenchyma is nevertheless mitigated in females if we consider the ability of SAG to exert negative effects on innate immunity in the EAE model. Thus, SAG induces a significant increase in NK cells, characterized as lymphocytes of the innate immune system that play a pivotal role in the defense against malignancies and viral infections. NK cells have recently emerged as a contributor to MS disease. On the one hand, the canonical CD56^dim^ NK cells, which are the most abundant in peripheral blood and the most well-known subtype, secrete IFN-γ, thereby creating a pro-inflammatory environment previously proposed to contribute to symptom exacerbation in MS by triggering the re-activation of the immune response [21]. On the other hand, the circulating CD56^bright^ NK cell subset corresponds to immunoregulatory cells in nature via their capacity to secrete distinct cytokines in response to chemical signaling. CD56^bright^ NK cells are able to produce IFN-γ and IL-10 alternatively depending on their environment and were proposed to play a protective role in autoimmunity [21]. The consistent SAG-induced increase in both pro- and anti-inflammatory cytokine levels detected in the lymph nodes from SAG-treated EAE female mice does not allow us to definitely identify the NK cell subset that is involved herein. In support of the involvement of the CD56^bright^ NK cell subset, SAG does not appear to display a deleterious effect in the strict sense in view of the decrease in the observed disability scores despite its inflammatory activity detected in females.

However, the dual effect of SAG may consistently account for the absence of the cooperation observed between SAG and testosterone in EAE females, which fully contrasts with data reported in EAE males. In the latter, the combination of SAG and testosterone led to the collapse of critical proinflammatory cytokines known to be associated with EAE pathogenicity, including GM-CSF, TNF-α, and IL-17A, in the spinal cord. Testosterone alone decreased IL-1β and TNF-α, while SAG failed to regulate any cytokines in males. Furthermore, testosterone was able to control peripheral immune cells in EAE males, more specifically by increasing CD4^+^ Foxp3^+^ regulatory T cells and the tolerogenic CD11c^+^ dendritic cells, which are both critical for the maintenance of immunological self-tolerance [9]. In females, testosterone alone or in the presence of SAG decreases the deleterious Th17 cells, likely resulting in a decrease in other pro-inflammatory cytokines, including SAG-induced cytokine production. We may thus summarize the causes and mechanisms leading to the main differences observed according to the sex of the animals by pointing out that, in females, the absence of testosterone results in an unexpected adverse effect of SAG on innate immunity, namely, on NK cells. This effect leads to the increase in inflammation in the lymphoid organs and, to a lesser extent, in the CNS, which likely contributes to modifying microglia response to demyelination if we consider the increase in microglial Arg-1 expression observed despite the higher level of myelination and the reduction in neurological disabilities. On the other hand, testosterone appears to display a beneficial effect on adaptive immunity, likely resulting in a specific activity in the immune context that makes it able to counteract SAG-induced inflammation. Conversely, in males, SAG has no effect on innate immunity in the absence of testosterone, while testosterone itself displays a beneficial effect on innate immunity. The synergistic activity of the SAG + T combination detected in males, unlike in females, leads to the collapse of several pro-inflammatory cytokines, mostly secreted by Th1 and Th17 cells, which is an effect that we have previously proposed to reflect a putative modulation of the well-known plasticity of these cells [9].

Previous works have proposed that the activity of the Hh signaling pathway influences the immune response, nevertheless without indicating if males or females were used. For instance, the overactivation of Hh signaling via the partial invalidation of the Patched transporter known to control Smo activity was found to predispose CD4^+^ T cells towards anti-inflammatory and neuroprotective Th2 polarization in the EAE model [22]. Along the same lines, the stabilization of EAE disease in Smo agonist-treated mice was correlated with decreased percentages of GM-CSF^+^ and IFN-γ^+^ GM-CSF^+^ CNS-infiltrating CD4^+^ T cells [23]. Finally, cell-autonomous Hedgehog signaling was found to control Th17 polarization and pathogenicity in mouse models of intestinal inflammation [24]. Now, we provide evidence for the sex-dependent effect of the Hedgehog signaling agonist SAG on the peripheral innate immune system, which specifically results in females with testosterone levels that are too low to cause an increase in peripheral inflammation, likely related to the activation of NK cells. Ultimately, this sex-dependent divergence conditions SAG’s ability to cooperate or not with androgens in the context of CNS demyelination. 

## Data Availability

The dataset is available on request from the authors.

## Supplementary Materials

The following supporting information can be downloaded at: https://www.mdpi.com/article/10.3390/cells13080676/s1, Figure S1: SAG and testosterone used alone or in combination increase Claudin-5 expression in EAE female mice; Figure S2: Representative dot plot showing sequential gating for identifying the main immune cell populations in the spleen; Figure S3: Representative dot plot showing sequential gating for identifying the lymphoid cell populations in the spleen; Figure S4: Representative dot plot showing sequential gating for identifying the main immune cell populations in the lymph nodes; Figure S5: Representative dot plot showing sequential gating for identifying the lymphoid cell populations in the lymph nodes.
