# Supplementary material for: The Smoothened agonist SAG Modulates the Male and Female Peripheral Immune Systems Differently in an Immune Model of Central Nervous System Demyelination
Source: Cells. 2024 Apr 13;13(8):676. doi: 10.3390/cells13080676 (PMC11048857; doi:10.3390/cells13080676)
Supplement: Supplementary file 1 [file cells-13-00676-s001.zip › cells-2905554-supplementary.pdf]

Kassoussi et al, Supplementary figures

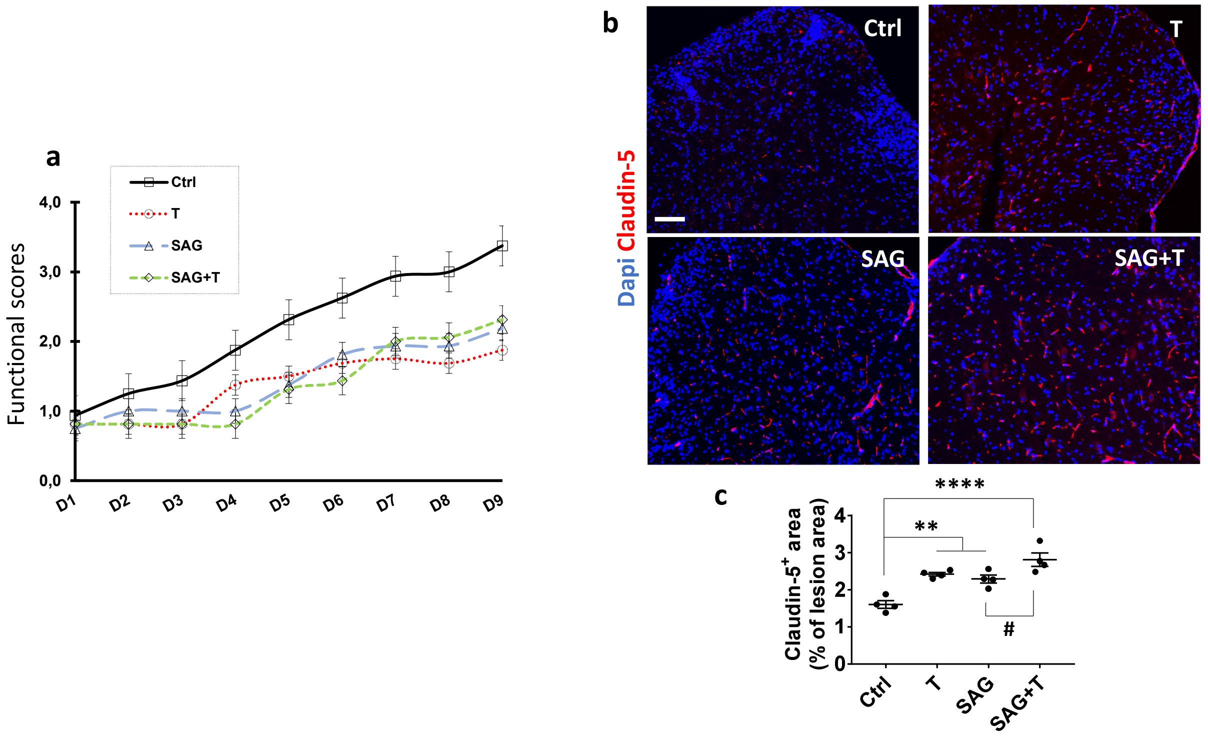

Figure S1. SAG and testosterone used alone or in combination increase Claudin-5 expression in EAE female mice. (a) Functional scores derived from EAE ovariectomized female mice treated with the drug vehicle (Ctrl), testosterone (T), the Smo agonist (SAG) or the combined drugs (SAG+T) from onset of the first neurological symptoms (day 1) until day 9. (b, c) At treatment day 9, Claudin-5 immunostaining and quantifications in the spinal cord. Each treatment condition increases Claudin-5. ANOVA tests are used for statistical analysis. ** $p \leq 0.01$; **** $p \leq 0.0001$; $^{\#}$ $p \leq 0.05$ compared to the indicated condition. Scale bar: 50 µm.

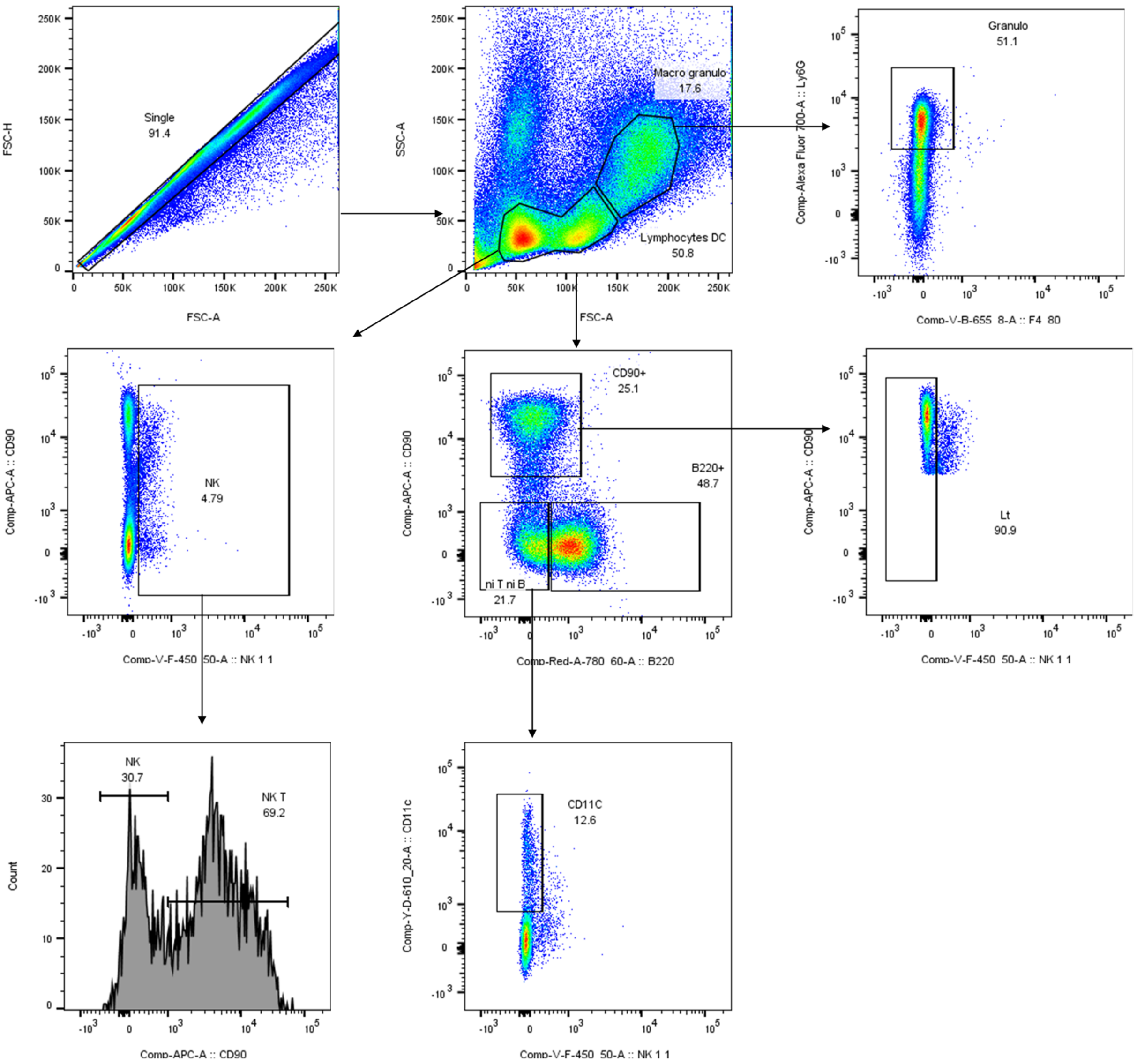

Figure S2. Representative dot plot showing sequential gating for identifying the main immune cell populations in the spleen. FSC and SSC parameters were used to select single, viable cells and exclude debris, dead cells and doublets. CD90 vs. B220 dot plot was then used to select $B220^{-}CD90^{-}$ non-T non-B cells and $B220^{-}CD90^{+}$ T cells. The $B220^{-}CD90^{-}$ non-T non-B cells were then gated by the expression of Ly6G (granulocytes), CD11c (dendritic cells), F4/80 (macrophages), NK1.1 (Natural Killer cells).

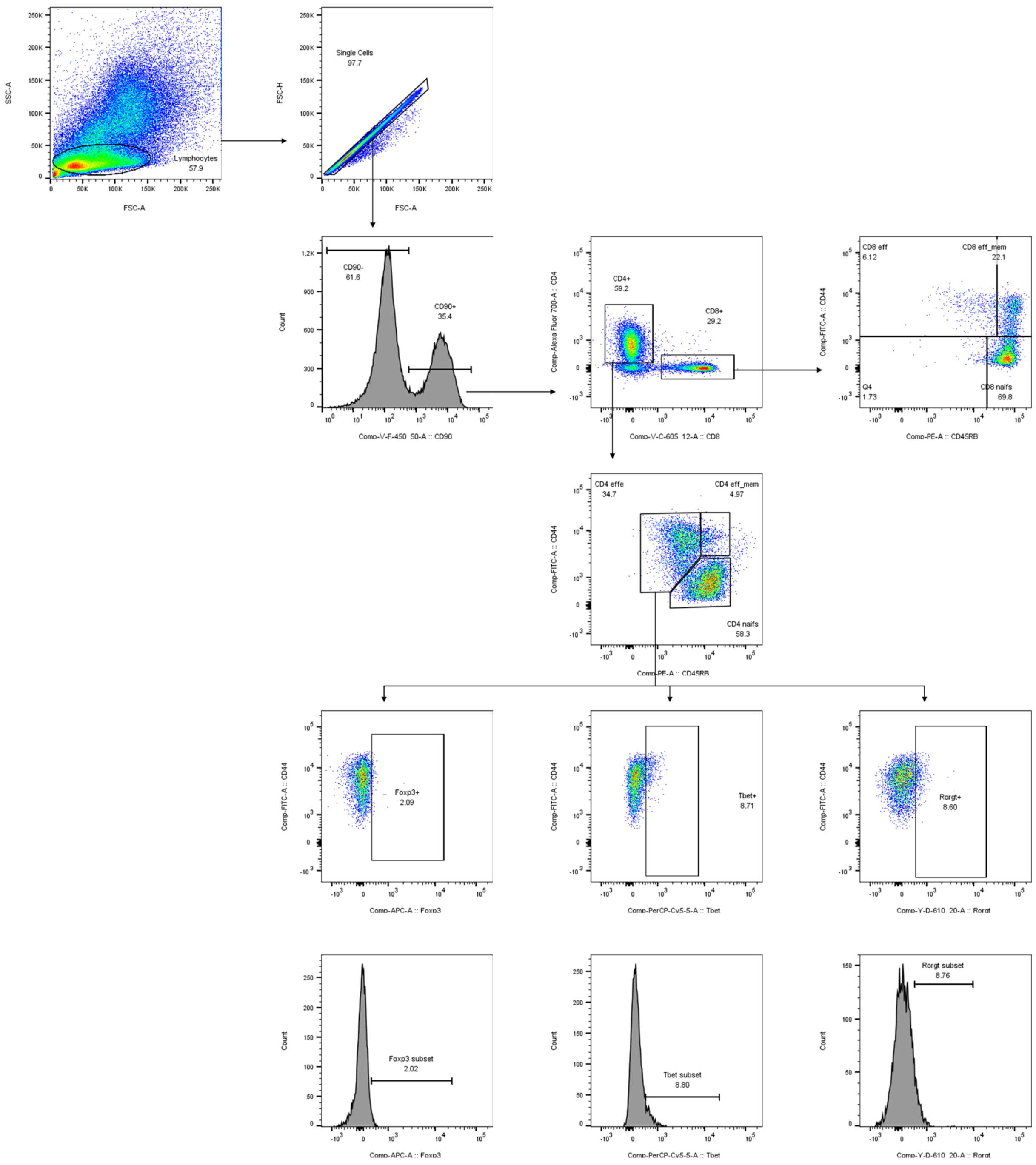

Figure S3. Representative dot plot showing sequential gating for identifying the lymphoid cell populations in the spleen. The CD90$^+$B220$^-$ T cells were gated by the expression of CD4. The relative expression of CD44 and CD45RB was analyzed on the CD4$^+$ T-cell subset to identify CD44^{lo}CD45RB^{hi} naïve and CD44^{hi}CD45RB^{lo} effector/memory cells. Finally, the percentages of cells expressing Foxp3, T-bet and RORγt were gated on CD44^{hi}CD45RB^{lo} effector/memory CD4$^+$ T cells.

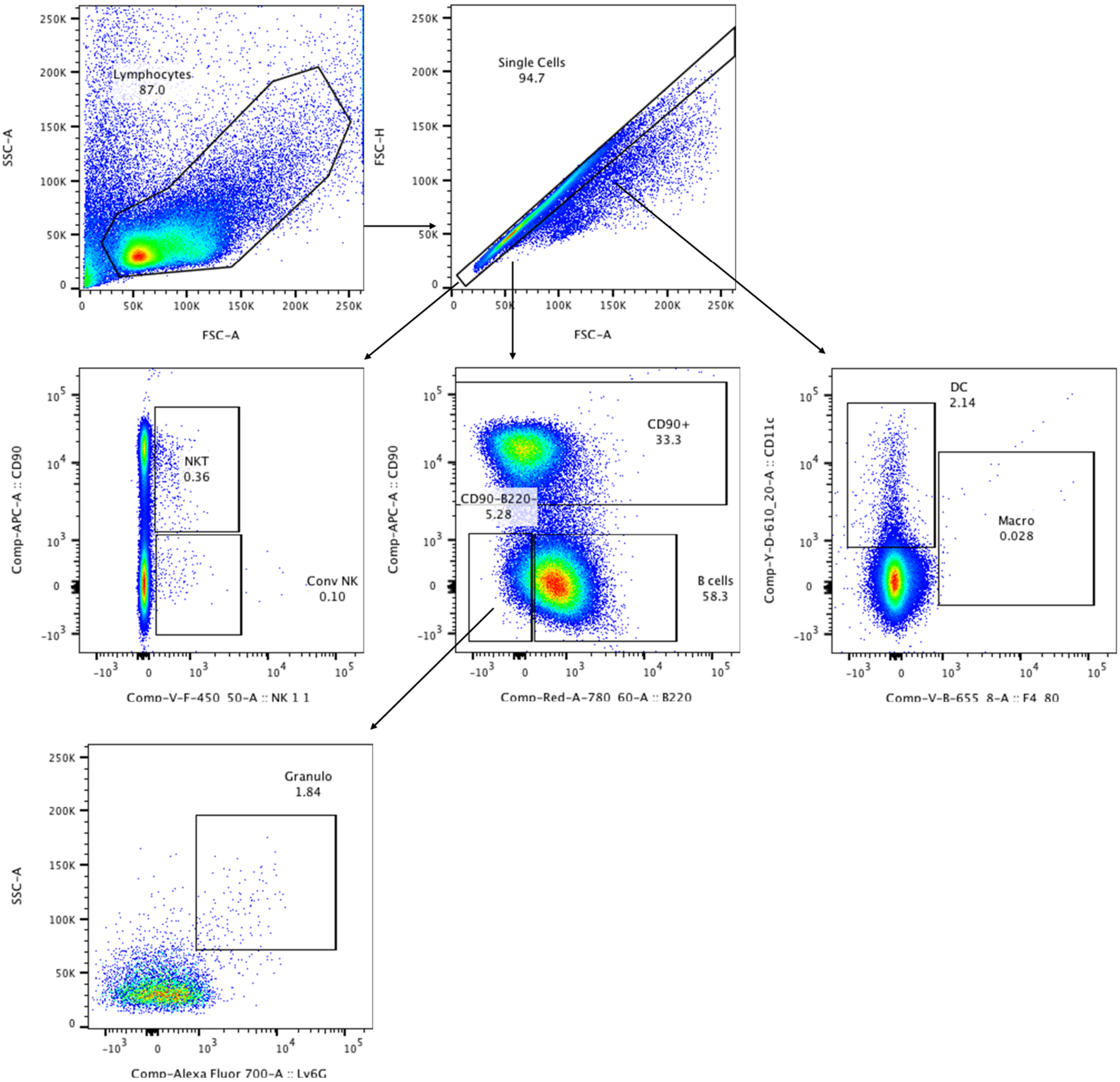

Figure S4. Representative dot plot showing sequential gating for identifying the main immune cell populations in the lymph nodes. FSC and SSC parameters were used to select single, viable cells and exclude debris, dead cells and doublets. CD90 vs. B220 dot plot was then used to select B220^{-}CD90^{-} non-T non-B cells and B220^{-}CD90^{+} T cells. The B220^{-}CD90^{-} non-T non-B cells were then gated by the expression of Ly6G (granulocytes), CD11c (dendritic cells), F4/80 (macrophages), NK1.1 (Natural Killer cells).

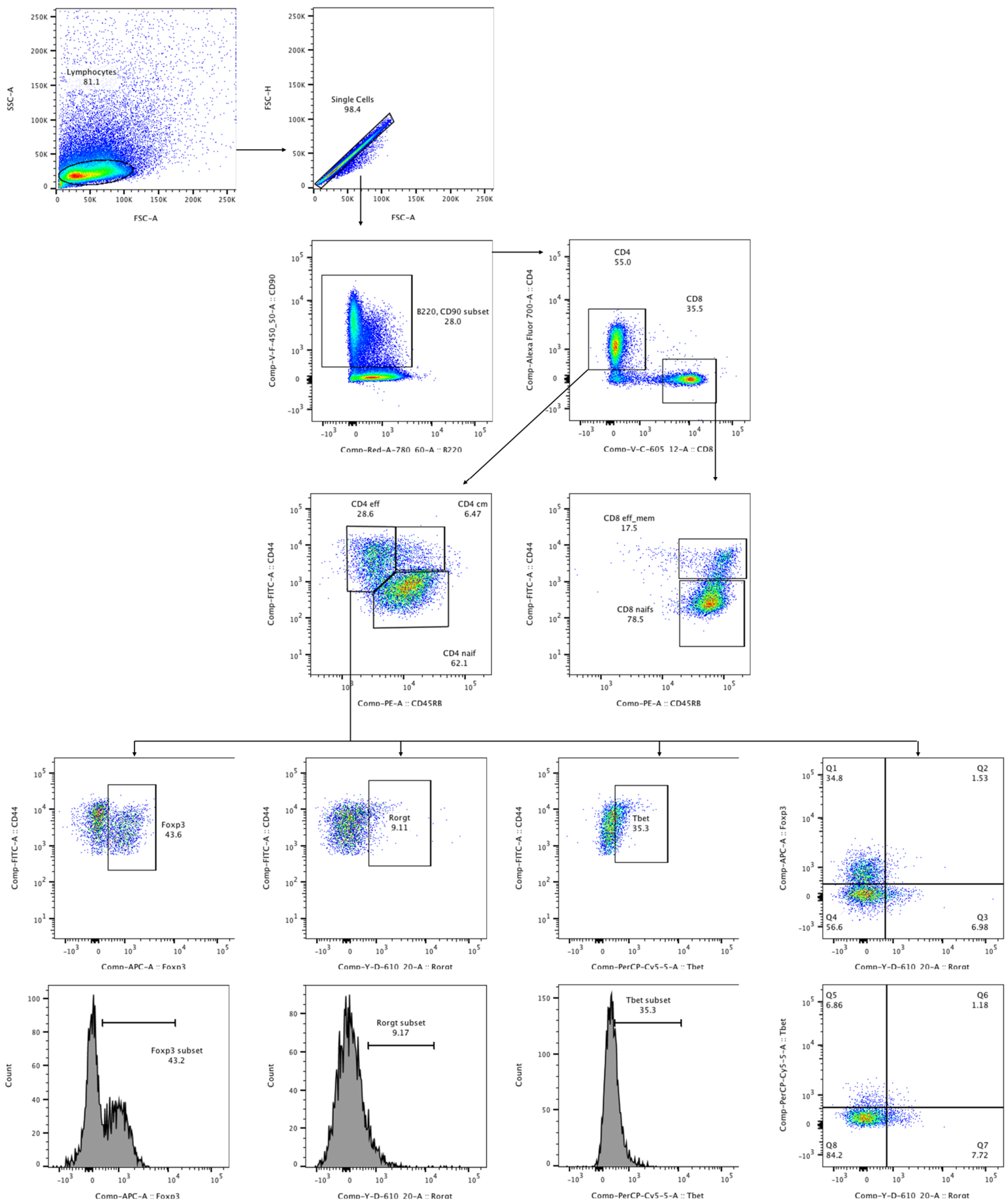

Figure S5. Representative dot plot showing sequential gating for identifying the lymphoid cell populations in the lymph nodes. The CD90$^+$B220$^-$ T cells were gated by the expression of CD4. The relative expression of CD44 and CD45RB was analyzed on the CD4$^+$ T-cell subset to identify CD44^{lo}CD45RB^{hi} naïve and CD44^{hi}CD45RB^{lo} effector/memory cells. Finally, the percentages of cells expressing Foxp3, T-bet and RORγt were gated on CD44^{hi}CD45RB^{lo} effector/memory CD4$^+$ T cells.
